# The column procedure preserves elbow stability on biomechanical testing

**DOI:** 10.1007/s00264-020-04494-0

**Published:** 2020-02-12

**Authors:** Andrzej P. Podgórski, Bartłomiej Kordasiewicz, Stanisław Pomianowski

**Affiliations:** 1 Department of Traumatology and Orthopaedic Surgery, Adam Gruca Clinical Hospital, Center for Postgraduate Medical Education, Otwock, Poland; 2 Department of Neurosurgery, Military Institute of Medicine, ul. Szaserów 128, 04-141 Warsaw, Poland

**Keywords:** Elbow stability, Elbow trauma, Elbow contracture, Column procedure

## Abstract

**Purpose:**

The effect of open release of a post-traumatic elbow contracture on the stability of the joint has not been so far studied in vivo. Resection of elbow joint capsule, the key element of surgery, was reported to have no effect on the stability of cadaveric elbows. The joint capsule is yet known to participate in maintaining elbow stability as one of secondary stabilizers.

**Methods:**

We assessed elbow joint laxity in 39 patients who underwent an open contracture release via the ‘column procedure’ described by B. Morrey and P. Mansat within the preceeding three to nine months. The measurements were taken with an apparatus designed particularly for this experiment according to the predetermined protocol. A preliminary part of the experiment showed that there was no significant difference between laxity of two elbow joints in healthy volunteers. Laxity of the operated elbows could be then compared with the contralateral joints.

**Results:**

Mean absolute difference of laxity between healthy and operated elbows was 1.55° (0.1°–4.1°, SD = 1.1) being significantly lower than 2°, *p* = 0.0056. The difference of the joint laxity between the operated and healthy elbows did not differ statistically significantly by more than 0.6° from the difference of the laxity of two healthy elbows and, therefore, is not clinically noticeable.

**Conclusions:**

Our experiment confirmed that the ‘column procedure’ is a safe procedure which does not compromise the stability of the elbow joint.

## Introduction

Elbow stability results primarily from the integrity of relevant anatomical structures which maintain physiological laxity of the joint. Laxity can be defined as range of motion of the joint in the coronal plane and differs broadly between individuals [1, 2]. Physiological amounts of elbow laxity provide stability of the joint, which is a clinically assessed feature and implies correct biomechanics of the joint. Following biomechanical testing of cadaver elbows, Nielsen [3] and Dos Remedios [4] independently concluded that resection of elbow joint capsule, which is the key element of surgery for a post-traumatic contracture, does not affect the joint laxity, hence would not lead to instability in clinical setting. Those findings have not been confirmed in vivo even though the joint capsule is reported to participate in maintaining the stability of the elbow [2]. In our study, we assessed elbow joint laxity in 39 patients who had undergone an open release of a post-traumatic contracture. The measurements were taken using a specially constructed apparatus which allowed full and safe immobilization of the upper limb and precise biomechanical testing.

Elbow stability derives from the congruence of the articular surfaces in roughly 50%, while the remaining half depends on the integrity of ligaments, capsule, interosseous membrane and, to a lesser degree, muscles of the arm and forearm which act as dynamic stabilizers [2, 5, 6]. Those structures are also classified as either primary or secondary stabilizers; the former are those whose injury leads directly to increased laxity of the joint, and the latter are the structures whose damage would increase laxity only after the relevant primary stabilizers had also been injured. Primary stabilizers include the anterior bundle of the medial collateral ligament, lateral collateral ligament complex and the congruence of the ulnohumeral joint [1, 7–9]. Important secondary stabilizers comprise the congruence of the radiohumeral joint, joint capsule and muscular attachments of the pronator and flexor muscles of the forearm and wrist to the medial humeral epicondyle and extensor muscles to the lateral epicondyle, respectively. An important part of the lateral collateral ligament complex, the lateral ulnar collateral ligament, serves as a primary stabilizer acting against posterolateral rotatory instability of the elbow [10].

Morrey and An in their biomechanical experiments examined the amount of contribution of the particular anatomic structures to the joint stability depending on the position of the elbow [2]. They noted that the articular capsule is responsible for 30% of valgus stability in full extension and that its importance diminishes with the flexion of the joint, dropping to near-zero values in 90° of flexion. The capsule also provides 32% and 13% of varus stability in elbow extension and 90° of flexion, respectively. It serves as the main stabilizer against joint distraction forces, providing 85% of resistance in extension and 8% in 90° of flexion. There is also an important relation between forearm rotation and elbow laxity, which is minimal in full supination, as demonstrated by Pomianowski [11].

The post-traumatic contracture of the elbow is a common complication of fractures and dislocations around the elbow caused by thickening and scarring of the articular capsule, altered shape and incongruence of bony surfaces, osteophyte formation, presence of intra-articular loose bodies and heterotopic ossification. Its occurrence is related to initial damage to the joint structures, intra-articular haematoma formation and individual propensity but can usually be effectively prevented by correct treatment and particularly by avoiding immobilization of the joint for longer than three weeks. Indications for surgical release include failure of physiotherapy after four to six months or presence of a surgical pathology, e.g. loose bodies within the joint, ossifications or a radioulnar synostosis. There are also patients with the so-called ‘stiff elbow’ who do not benefit from physiotherapy and who should be treated surgically without the usual delay.

Our experience involves 279 patients who have undergone an open release of post-traumatic elbow contracture in our centre since 2003 (Table 1). The relatively high percentage of patients who developed a contracture following a radial head fracture was mainly due to prolonged immobilization of the joint and lack of adequate physiotherapy. Our surgical technique follows the outline of the ‘column procedure’ described by B. Morrey and P. Mansat [12]. The joint is approached via the anterolateral route between the extensor carpi radialis longus and brevis and the extensor digitorum communis anteriorly to the lateral collateral ligament. The articular capsule is bluntly dissected and excised until the anterior aspect of the ulna is visualized. The posterior compartment of the joint can be opened through the same incision if needed, and posterior capsulectomy can also be performed. Scar tissue, osteophytes and intra-articular loose bodies are removed. If deemed necessary, the procedure can involve other steps, e.g. radial head excision, interpositional arthroplasty, excision of synostosis or ulnar nerve transposition via a medial approach. Although there is no agreement on the superiority of the radial head replacement over simple excision [13], we always replaced the radial head with a prosthesis (KPS) designed by the senior author. Release of the ulnar nerve was consistently combined with its anterior transposition and was never performed prophylactically despite recent reports underlining its benefit [14–16]. Arthrolysis of the elbow can be performed using other approaches, most often Kocher approach, as well as combined with exposing the joint from the medial or posterior aspect [17–20] depending on the underlying pathology [21]. Sequelae of complex injuries, e.g. terrible triad of the elbow, are particularly demanding. Protocols involving extensive arthrolysis, radial head excision and temporary external fixation have been elaborated [22, 23]. Due to the complexity of elbow injuries, optimal treatment of their sequelae varies among different centres according to the individual experience [23–25].

**Table 1:** Initial lesion of the elbow in patients who subsequently underwent an open release of its contracture in our series

Lesion	Number of cases	Percentage
Distal humeral fracture	58	20.8
Radial head fracture	62	22.2
Proximal radius and ulna fractures	13	4.7
Elbow dislocation	18	6.4
Elbow dislocation and radial head fracture	19	6.8
Elbow dislocation and other or multiple fractures	20	7.2
Olecranon fracture	16	5.7
Radius and ulna shaft fractures	2	0.7
Multiple or other fractures	35	12.5
Trauma to elbow with no fracture	36	13.0
All	279	

The results of the operative treatment vary from excellent to poor being closely dependent on the pre-operative status of the elbow, namely, on bone alignment and articular incongruence, as reported in other series [26–28]. An analysis of the 213 cases in which full medical record was available including the final assessment 12 months after the operation showed that mean MEPI score following surgery was 86.3 points compared to 63.2 points preoperatively (mean gain 22.9 points). If the articular surfaces were intact or healed in anatomic alignment, the results were usually spectacular with a significant operative gain regarding both the range of motion and the antalgic effect. Mean gain in flexion-extension range of movement after 12 months was 29° (− 10 to 95°), and mean improvement of forearm rotation was 26.8° (0 to 140°). In 27 cases, the surgical procedure involved other steps listed in Table 2. Seven patients required re-operation due to recurrence of the contracture, and two patients developed a deep infection.

**Table 2:** Additional procedures performed during the surgical release of elbow contracture in our patients

Radial head replacement (KPS bipolar endoprosthesis)	9
Radioulnar synostosis resection	6
Lateral collateral ligament repair	12

## Materials and methods

Assessment of elbow joint stability was performed using an apparatus designed particularly for this experiment, which measured the laxity of the joint by pivoting the forearm of the immobilized upper limb into valgus and varus alignment. The combined deviation angle was subsequently calculated by the device and was established as the physiological laxity of the examined elbow joint.

**Fig. 1:**
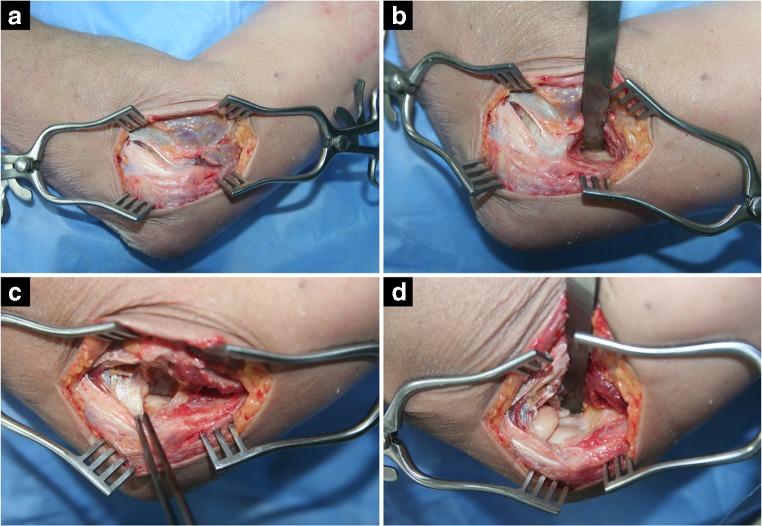
Technique of ‘column procedure’. **a** Development of interval between the extensor carpi radialis longus and brevis and the extensor digitorum communis. **b** Blunt retractor is passed exposing anterior surface of distal humerus. **c** Exposure and incision of lateral capsule. **d** Final operative view after anterior capsulectomy and exposure of coronoid process

**Fig. 2:**
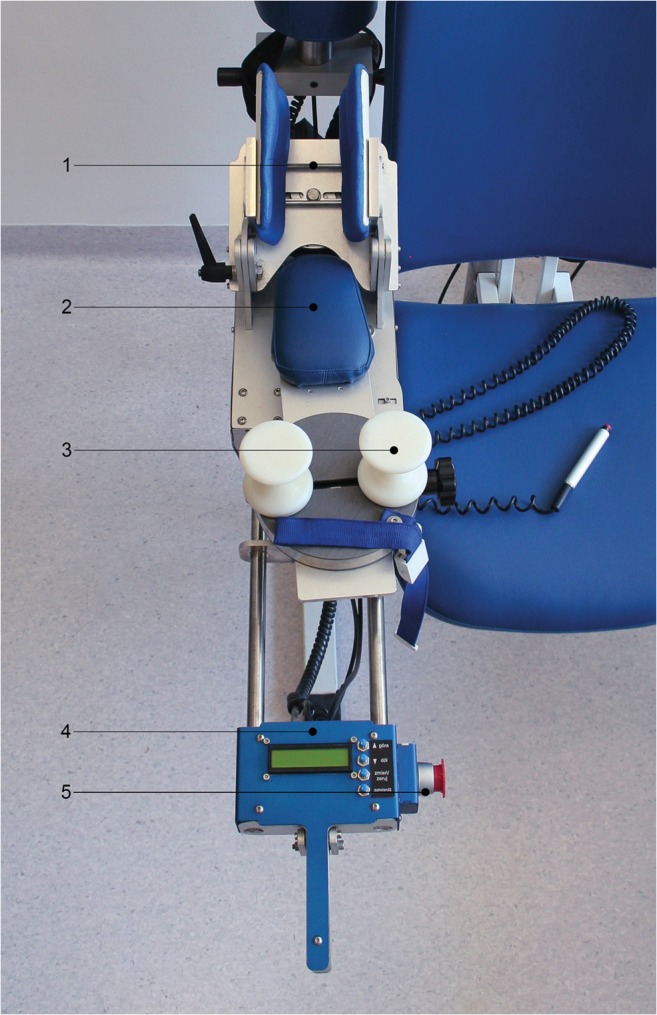
The adjustable frame of the measuring device was equipped with a padded arm holder (1), elbow support (2), wrist immobilizer (3), electronic module (4) and safety switch (5) allowing steady and comfortable positioning of the examined limb. See text for details

**Fig. 3:**
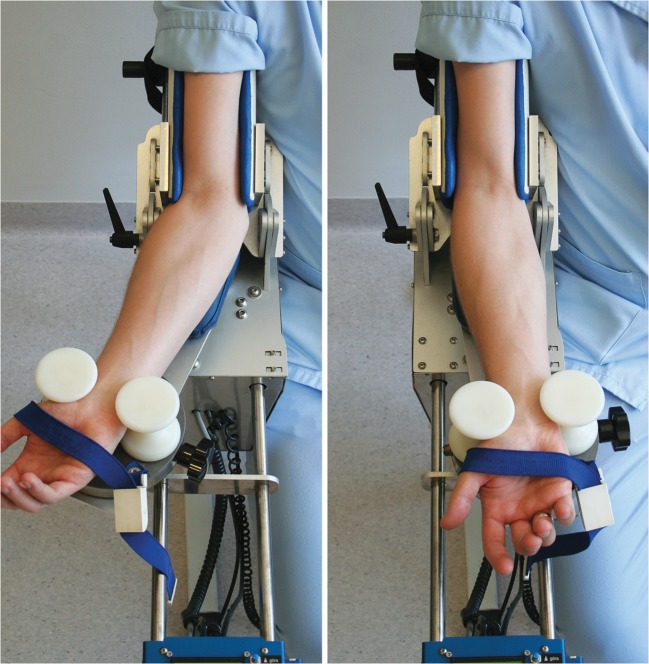
The measuring device conducted a cycle of transitions of the forearm between maximal valgus and varus deviations according to the predetermined protocol

The apparatus consisted of a chair equipped with an extending arm and a moveable frame containing the elbow and wrist immobilizers, the measuring module and the control panel (Figs. 1 and 2). The examined limb was immobilized in a padded two-part holder covering the distal part of the arm above the humeral epicondyles, which was tightened after the limb had been placed in the correct position. Padding increased the comfort of the patient and prevented the arm from rotating in the holder. The axilla was additionally fixed by an adjustable-length strap. The tested limb was positioned in 30° of elbow flexion to increase the contribution of the joint capsule to the stability of the elbow. The distal part of the forearm and the wrist was blocked in full supination to reduce the inherent laxity of the joint, as we assumed that any increase in the laxity would be more evident in this position (Fig. 3).

**Fig. 4:**
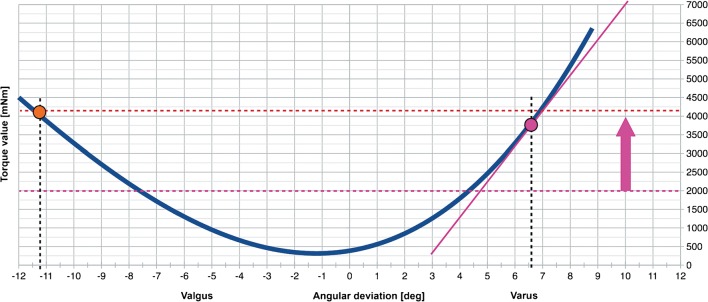
Illustrative graph showing mean relation between torque values exerted by the measuring device and the forearm deviation. Two dotted horizontal lines represent minimal (lower) and maximal (upper line) torque values predetermined by the investigators. Vertical arrow shows range of torque values within which measurements could be registered. Two coloured dots show mean valgus and varus deviation value. It can be appreciated that the valgus dot is located on the maximal torque line, while the varus dot is slightly lower. This shows that whereas the valgus deviation was registered upon reaching maximal torque value, the varus deviation was recorded when maximal torque derivative was reached

The maximum valgus and varus forearm deviation was recorded based on the analysis of the increase in the moment of resistance. The measuring module recorded the current angle of the forearm deviation and the instantaneous torque to monitor the value of the torque derivative (Fig. 4). Deflection of the forearm would be stopped when either the torque itself or torque derivative reached the maximum value defined by the investigators. These values had been predetermined experimentally to ensure that the forearm was deflected strongly enough, but the applied force did not cause the pain or arm movement in the immobilizing holder (Fig. 3). Minimum value of the torque derivative was also adjusted, which prevented the position of the maximum deviation from being recorded too early, e.g. due to muscle tightening by the examined patient. The selected parameters were identical for all measurements in the entire study.

According to the established protocol, the measuring apparatus performed a cycle consisting of six transitions from maximum valgus to maximum varus deviation. At each extreme position, the angular value was recorded for the difference between neutral position and extreme deviation. Two results – the highest valgus and varus deviations – were rejected. The remaining values were used to calculate the mean joint laxity, being the sum of the mean valgus and varus deviations. High reproducibility of results was obtained; the difference between the results rarely exceeded 0.5° for individual deviations in the same direction.

The aim of our study was to determine if a correctly performed ‘column procedure’ leads to an increase in the physiological laxity of the joint, which in turn could result in instability of the elbow. In our reasoning, we assumed that the laxity of the joint following the operation could be assessed by comparing it with the contralateral normal elbow. The goal of the preliminary part of the experiment was therefore to define if there is any difference in laxity between the two elbow joints in a healthy individual. A series of measurements was taken on a group of volunteers recruited from 52 healthy individuals (29 males, 23 females) of mean age of 34 years (24 to 68) [29]. The selection criteria were normal anatomy and function of both elbow joints with no history of trauma to upper limbs or rheumatologic disease. Owing to the construction of the measuring apparatus, also individuals of exceptionally sturdy or slender build as well as those in whom their natural valgus angle of elbows exceeded 20° could not participate in the study. The results led to a conclusion that although the range of physiological elbow laxity defined as the deviation from the maximal valgus to maximal varus position varies significantly (10.6°–26.5°, mean value 17.8°), the difference in the laxity between two elbows of the same volunteer was only slight, amounting to a mean value of 1.19° (0.1°–3.8°). Moreover, there was no correlation between the side of the elbow with greater laxity and the dominant limb. We therefore concluded that the contralateral elbow could be used as a reference to assess a possible change of the joint laxity following surgery.

The main part of the experiment consisted of examination of 39 patients who had undergone a standard open release of a post-traumatic elbow contracture in our centre during the preceeding 12 months. The patients were recruited according to precise criteria (Table 3).

**Table 3:** Inclusion and exclusion criteria for biomechanical testing of the elbow stability in operated patients

Inclusion criteria	
1. Standard open release of a posttraumatic elbow contracture (‘column procedure’)	
2. Surgery within the preceeding 3 to 9 months	
Exclusion criteria	
1. Permanent damage to articular surfaces of the elbow	
a. Malunited intraartricular fractures	
b. Defect of an articular surface, e.g. excision of the radial head	
c. Radial head replacement	
2. Surgical repair of a collateral ligament	
3. Patients undergoing multiple surgeries for a contracture	
4. Injury or other pathologies involving the contralateral elbow (except nerve entrapment or asymptomatic tendinopathy)	
5. Technical limitations or problems with adhering to the protocol	
a. Patients of particularly sturdy or slender build	
b. Persistent flexion contracture limiting elbow extension to more than 30°	
c. Individual valgus alignment of elbow of more than 20°	

The measurements took place after completion of the physiotherapy and reaching the optimal range of motion of the elbow. The proposed time frame of three to nine months between the surgery and the examination was short enough to ensure that the results would not be influenced by any degenerative changes in the joint.

There were 39 patients of mean age of 33 years included in the study, 25 (64.1%) of whom were males and 14 (33.9%) females. Thirty-six patients (92.4%) were right-handed, and three patients were left-handed (7.6%). The most prevalent injuries to the elbow were radial head fracture (11 cases – 28.2%) and distal humeral fracture (9 cases – 23%), which was in accordance with our overall experience.

The mean flexion and extension of the elbow joint before the surgery were 117.4° and 44°, respectively, compared to 125.7° and 19.4° after the surgery. The resulting mean increase in the range of motion with respect to the flexion and extension arc was 32.8°. The mean increase in the rotational movements of the forearm was 13°. The average MEPI score was 67 points pre-operatively and 91 points post-operatively. In 24 patients (61.5%), the greater range of physiological laxity was found in the intact elbow joint and in 15 (38.5%) patients in the operated joint.

## Results

For each patient, total range of laxity was calculated separately for the healthy and operated elbow. The absolute difference between the laxity ranges of both elbows was then calculated. As stated earlier, analogous data had been previously obtained in healthy volunteers.

Wilcoxon signed-rank test was used to verify the hypotheses concerning mean values of absolute differences between healthy elbows and between healthy and operated elbows by comparing the mean differences with the values of 1° and 2°. The equality of the absolute differences in the laxity of elbows between the healthy and operated groups was checked using the Mann-Whitney U test. Statistical significance was assumed at *p* < 0.05. The difference of 1° between laxity of the two elbows was considered as clinically noticeable.

The results of previously performed examinations assessing range of laxity in healthy subjects showed that the mean elbow valgus deviation was 11.2° (6.4°–16.1°) and mean elbow varus deviation was 6.6° (3°–10.7°). The laxity range of the elbow was 17.8° (10.6°–26.5°). The mean difference in laxity between the two opposite elbows in the same person was 1.19° (0.1°–3.8°, SD = 0.84) and was significantly lower than 2° (*p* < 0.0001). It should be noted that there is no correlation between the side with greater elbow laxity and the dominant side. Although there were 94.2% right-handed patients in the healthy group, only in 57.6% of the volunteers the right elbow was the joint with a greater range of motion in the coronal plane than the left one.

The results of the study in patients after surgical release of elbow joint contraction indicated that the mean absolute difference in the laxity range between healthy and diseased elbows was 1.55° (0.1°–4.1°, SD = 1.1) and was also significantly lower than 2°, *p* = 0.0056.

The comparison between the two groups showed that the difference in the elbow laxity range between the operated and healthy elbows in the operated patients did not differ statistically significantly from the difference in the elbow laxity range between two healthy elbows in the healthy group (*p* > 0.1).

The collected data allowed for detection of a difference of 0.3° in the healthy group and 0.46° in the group of operated patients with a statistical power of 80%. With 52 and 39 observations in the healthy and operated groups respectively, the mean value of 1.19° in a group of 52 healthy volunteers and the standard deviation in both groups of 1°, a difference between means of 0.6° could be considered as statistically significant. It has been found that the difference in the total deviation between the operated and healthy elbows does not differ statistically significantly by more than 0.6 from the difference in the total deviation of the two healthy elbows and, therefore, is not clinically noticeable.

## Discussion

Column procedure is a well-established and efficient technique in elbow surgery associated with favourable results. Although joint instability is not recognized as a common complication of this type of surgery, this issue has received little attention. The aim of our study was to analyse in an objective manner whether column procedure affects elbow laxity, which could in turn lead to joint instability. The vast experience of our centre allowed us to operate in a reproducible manner and to obtain surgical results concomitant with those of other authors, which additionally validates our conclusions.

According to our knowledge and thorough search through the available medical databases, there were only two reports involving similar studies conducted in cadaver labs. Nielsen et al. examined the effect of anterior and posterior capsulectomy on elbow laxity in seven cadaver specimens, finding no convincing influence. Dos Remedios et al. in another study came to the same conclusions. These experiments were not, however, continued in vivo, and we suppose that the clinical value of these studies was limited as some of the biomechanical aspects, e.g. dynamic forces exerted by muscles around the elbow, could not have been well reproduced. As far as we are concerned, our experiment was the first biomechanical assessment of elbow laxity following column procedure with the use of a dedicated measuring device.

The main limitation of our study is its narrow field. The study was not designed to analyse operative results of a widely used procedure nor to present improvements to the surgical technique but to prove using an impartial approach and sophisticated equipment that joint laxity is not violated.

## Conclusion

Our experiment confirmed that the ‘column procedure’ is a safe procedure which does not compromise the stability of the elbow joint.
